# Management of Bleeding, Thrombotic and Pregnancy-Related Complications in Women with Myeloproliferative Neoplasms: A Case-Based Review Focusing on Sex-Specific Challenges

**DOI:** 10.3390/jcm14051537

**Published:** 2025-02-25

**Authors:** Thita Chiasakul, Ross I. Baker

**Affiliations:** 1 Center of Excellence in Translational Hematology, Division of Hematology, Faculty of Medicine, Chulalongkorn University and King Chulalongkorn Memorial Hospital, Bangkok 10330, Thailand; thita.c@chula.ac.th; 2 Western Australia Centre for Thrombosis and Haemostasis, Health Futures Institute, Murdoch University, Perth, WA 6150, Australia; 3 Clinical Research Unit, Perth Blood Institute, Perth, WA 6005, Australia

**Keywords:** myeloproliferative neoplasm, thrombosis, bleeding, women, pregnancy

## Abstract

Myeloproliferative neoplasms (MPNs) are a heterogeneous group of clonal hematopoietic disorders that pose unique challenges in women, particularly regarding thrombosis, bleeding, fertility, and pregnancy. Women with MPN exhibit distinct thrombotic and sometimes contradictory bleeding profiles, including a higher prevalence of unusual thrombosis such as cerebral and splanchnic vein thrombosis and increased risk of hemorrhage from anti-thrombotic medication, acquired von Willebrand syndrome and platelet dysfunction. Estrogen-containing contraceptives should generally be avoided due to thrombotic risk. Around 10–20% of newly diagnosed MPN cases are women of childbearing age and the number is increasing annually. MPN patients when compared to controls have a lower rate of live birth rate of 71% vs. 80% with a hazard ratio of 0.78 (95% CI: 0.68–0.90), and increased preterm birth (14% vs. 4%), low birth weight (<2500 g, 10% vs. 4%), and increased cesarean section rate (32% vs. 17%). Management of MPN-related pregnancy requires specific considerations regarding the prevention of thrombosis, bleeding, and pregnancy-related complications. Management strategies during pregnancy include low-dose aspirin and consideration of low-molecular-weight heparin and interferon. Despite these challenges, most women with MPN can achieve successful pregnancies with optimized care. In this case-based review, we present two cases that illustrate key aspects of managing MPN in women, summarize the current literature, and propose a diagnostic and management framework tailored to these complexities.

## 1. Introduction

The classical Philadelphia chromosome-negative myeloproliferative neoplasm (MPN) is a heterogeneous group of clonal hematopoietic stem cell disorders that include polycythemia vera (PV), essential thrombocythemia (ET), and primary myelofibrosis (PMF) [1]. These disorders are characterized by one of the shared driver mutations, *JAK2*, calreticulin (*CALR*), and *MPL*, which result in consecutive activation of the JAK-STAT signaling pathway, eventually leading to abnormal hematopoietic cell proliferation and excessive cytokine production [2]. Thrombosis and bleeding are common complications that contribute to significant morbidity and mortality in MPNs [3,4]. Consequently, the prevention of thrombotic events is considered one of the primary goals in the management of MPN and therapeutic options are considered based on an individual’s thrombotic and bleeding risk profile [5,6]. Pregnancy represents a heightened pro-thrombotic state and when compounded by MPN, the risk escalates further, presenting considerable challenges to fetal and maternal outcomes [7].

Despite sharing similar genetic mutations, significant heterogeneity exists in the epidemiology, clinical phenotypes, and prognosis among the MPN entities, underscoring the wide spectrum of disease and the important roles of other biological modifiers [3,5,6,8,9,10]. While age and thrombotic history are the more recognized factors influencing MPN characteristics, sex is also an important determinant of the natural history and prognosis of MPN as outlined in Table 1 [11].

**Figure 1:**
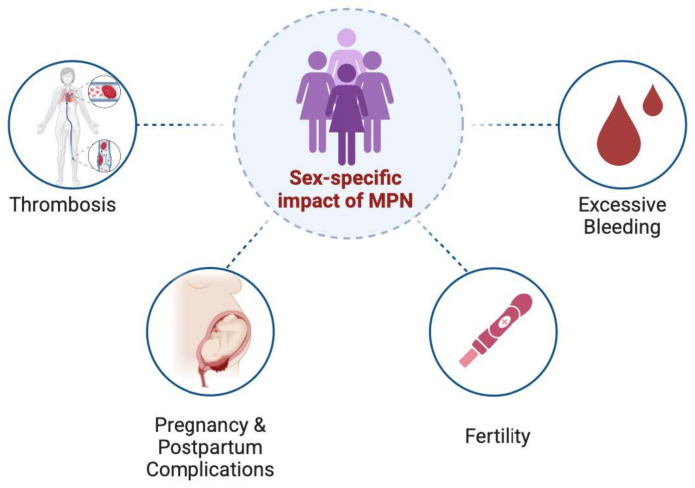
Impact of myeloproliferative neoplasms on women’s health. MPN: myeloproliferative neoplasm.

Although the standard of care for MPN remain similar across sexes, managing women with MPN poses several specific challenges unique to women’s health as shown in Figure 1.

One unique issue is heavy menstrual bleeding (HMB), which can be exacerbated by the bleeding diathesis associated with MPN, often linked to acquired von Willebrand syndrome (aVWS) [16]. HMB can also reduce tolerance to phlebotomy, complicating standard treatment approaches. While controlling HMB is a priority, the use of combined estrogen-containing contraceptive pills is generally discouraged due to their association with a 3-fold increase in thrombotic risk [17]. Thrombosis presents another critical concern, as women with MPN do face heightened thrombotic risks, particularly during periods of hormonal fluctuation such as pregnancy or the use of hormone-based therapies [7]. Finally, questions surrounding the impact of MPN and its treatments on fertility and pregnancy outcomes are common among women of reproductive age [7]. These concerns necessitate a comprehensive team approach to care, including pre-conception counseling, close monitoring during pregnancy, and individualized management strategies to optimize both maternal and fetal health.

In this case-based review, we present two illustrative cases that highlight key aspects of managing MPN in women and propose a diagnostic and management framework tailored to address these complexities.

## 2. Bleeding in Women with MPNs

**Case Presentation 1:** A 39-year-old woman with recurrent iron deficiency was referred for investigation of an abnormal CBC and possible underlying bleeding disorder. She has a history of HMB arising since the birth of her first child 5 years ago. Her periods typically lasted 8 days, requiring regular pad changes (every 2 h) for the first 3 days impacting her attendance at work. In addition, she has required four intravenous iron infusions over the last year. Previously, her HMB has been refractory to the OCP and subsequently required dilation and curettage and insertion of a progesterone-only intrauterine contraceptive device, which also has been ineffective. She also describes excessive bleeding following four molar extractions 1 year previously that necessitated hospital readmission, packing and resuturing. Finally, she recently describes increasing easy and more extensive bruising. There is no family history of a bleeding disorder, and she is on no medications. Her CBC reveals an HB of 120 g/dL, WCC of 8.0 × 10^9^/L and platelets of 800 × 10^9^/L with microcytosis (MCV 78fl) on blood film.

### 2.1. The Challenge of Assessment of Bleeding in MPNs

Hemorrhage is a recognized complication of MPNs, with the incidence of major bleeding (MB) in ET in the range of 0.43–5.3 events and for PV 0.3–5.3 events per 100 patient years [6,8,18,19,20]. Two large retrospective studies of 1545 patients with PV and 1104 patients with ET and PMF consistently showed that MB occurs in 4.2%, 6% and 12% of MPNs, respectively (PV/ET/PMF), during a median follow-up period of 6.2–6.9 years [21,22]. The most common site for bleeding is the gastrointestinal tract followed by the urogenital system, other mucocutaneous systems and the central nervous system [20]. With MB, there is a significant case fatality rate ranging from 3.1 to 26.7% [20].

Excessive bleeding with MPNs is an important sex issue because around 10–20% of patients with newly diagnosed MPNs are women of childbearing age [19,23]. For this reason, MPNs are a rare but not uncommon cause of HMB and post-partum haemorrhage, which are reported but likely to be underestimated in larger cohort studies using conventional bleeding severity definitions [7,24]. Furthermore, in MPN stuides, the significant negative impact of quality of life of HMB is not measured and likely to not be disclosed because of the stigma and cultural issues associated with menstruation as well as the lack of healthcare provider awareness and dismissal of symptoms [25]. The availability of testing for myeloid genomic sequencing variants for driver MPN mutations (*JAK2* V617F, *CALR* and MPL) when MPN is suspected is likely to increase the diagnosis of MPN’s in younger patients. In a population-based study from 2000 to 2014, the crude incidence of all MPNs under 49 years of age was 2.32/100,000 person years, with ET being the most common MPN subtype in women, and the incidence rate is increasing each year [19].

There are varying expert opinions on guidance for bleeding management in MPNs, mainly because of the low level of evidence arising from retrospective cohort studies or case reports. No prospective studies utilizing standardized MPN inclusion criteria and timely laboratory testing for bleeding risk stratification have been undertaken, especially prior to surgery or when a bleeding episode occurs. Consequently, the evidence base for understanding MPN bleeding is inconsistent with a high risk of bias, which leads to a wide variation in published studies of the many factors that could increase bleeding risk. These include the distribution of MPN subtypes, clinical stage, *JAK2* V617F allele burden, duration of disease, the use of antithrombotic or cytoreductive treatment and other non-clinical MPN risk factors.

### 2.2. Risk Factors for MPN Bleeding

**Figure 2:**
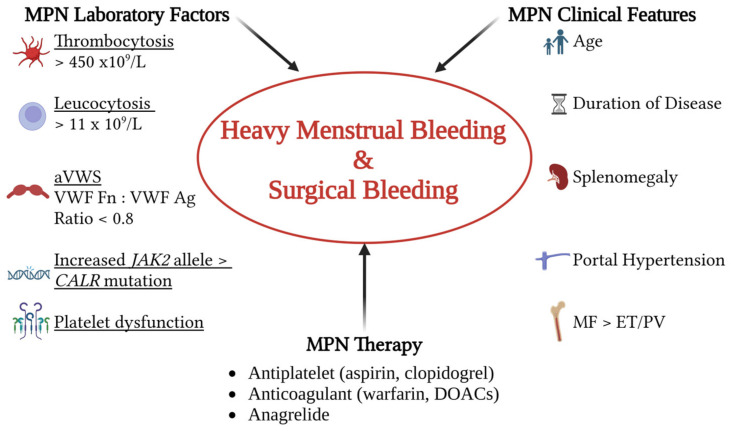
Clinical and laboratory factors to consider in the assessment of bleeding risk for women of child-bearing age with MPNs [21,22,24,26,27,28,29,30,31]. HMB and risk of excess bleeding with surgery are often underestimated issues for clinical practice. MPN, myeloproliferative neoplasm; MF, primary myelofibrosis; ET, essential thrombocythemia; PV, polycythemia vera rubra; VWF Fn, assay that measures VWF activity; VWF: Ag, VWF antigen; VWF, von Willebrand Factor; *JAK2*, *JAK2* V617F allele burden; *CALR*, calreticulin gene mutation; DOACs, direct oral anticoagulants; aVWS, acquired von Willebrand syndrome.

The risk factors to consider for excessive bleeding risk in MPN patients are multifactorial as shown in Figure 2. Evidence for association is not strong and inconsistent with different studies, so risk factor appraisal may assist rather than be a definitive answer for practical decisions on the management of bleeding in MPNs. A personal history of bleeding is the most relevant assessment for a clinican to guide individual management. A platelet count above the normal range or leukocytosis have been shown to be correlated with major hemorrhage [21,26]. Studies on the *JAK2* V617F allele burden have been conflicting with one study showing association between those with the highest 4th quartile level with bleeding risk (IR ratio 4.6) whilst another did not [27,28]. Although the CALR mutation is associated with increased platelet count when compared to the *JAK2* V617F allele, the presence was not associated with increased bleeding risk [29]. Those ET patients with the highest risk of thrombosis as measured by the IPSET-t score (based on age, thrombosis history, *JAK2* V617F mutational status and cardiovascular risk factors) also had a paradoxical 4.2-fold increased bleeding risk (*p* = 0.003) when compared to lower risk categories [30]. However, another study with shorter followup could not confirm this finding [31]. Unexpectedly, diabetes mellitus has recently been identified as a novel risk factor for ET-related bleeding (HR 4.12; 95% CI, 1.17–13.52), which was speculated because of the known association of diabetes with impaired wound healing [24]. This finding needs confirmation in further additional studies.

There is an interaction of the clinical factors, some of which are MPN-specific, that could exacerbate other known non-MPN risk factors for bleeding, such as unrelated GIT pathology, uncontrolled hypertension, impaired renal or liver function, and use of non steriodal anti-inflammatory agents [24]. Laboratory testing often reveals abnormalities of hemostasis such as aVWS or quantitative or qualitative disorders of platelet function [32]. Anti-thrombotic medication (such as aspirin, clopidogrel, warfarin or direct oral anticoagulants [DOACs]) or thrombocytopenia caused by excessive cytoreductive treatment or part of the progression of the MPN will also increase bleeding risk especially around the time of surgery [31].

As MPN is not a static disease, there is evidence to suggest that the bleeding phenotype can change over time [33], especially with the acquisition of nondriver mutations that are associated with an increased bleeding risk such as DNMT3A (hazard ratio 6.09; CI 1.44–25.48) reported to occur in 4% of ET patients [24]. The known transformation of PV and ET to secondary myelofibrosis and acute myeloid leukaemia is also likely to change the bleeding phenotype but this has not been systematically studied.

### 2.3. Mechanisms for Bleeding

The biological cause of the bleeding tendency in MPN is also multifactorial. aVWS has been frequently reported in MPN and associated with excess bleeding caused by the loss of the hemostatically active high molecular von Willebrand factor (VWF) multimers (HMWM) [16,34,35]. The mechanism is thought to be increased proteolysis of VWF mediated through cleavage by the ADAMTS-13 enzyme [36] or increased absorption of the VWF on platelets and removal from the circulation [16,34,35]. It is often but not always corrected by MPN cytoreductive therapy [16,34,36,37]. aVWS can occur with any platelet count but particularly in patients with extreme thrombocytosis of >1000 × 10^9^/L [38,39,40]. The prevalence varies according to the diagnostic definition with some studies reporting over 50% of MPN cases have aVWS and that MPN is correlated to increasing platelet and leucocyte counts [35,36,41]. The diagnosis of aVWS is challenging because the values of the VWF activity (VWF:Act) to VWF antigen (VWF:Ag) ratio are not necessarily less than normal [16,35,41]. Instead, patients with aVWS have decreased VWF:Act to VWF:Ag ratios, but studies use different cut-off ranges for diagnosis, which vary between <0.6 and 0.8 [16,34,35,36,41]. However, most patients with aVWS do not seem to bleed even whilst on aspirin [24], but no studies have examined whether aVWS has an impact on triggered bleeding with surgery. Perhaps, until further data are available, a more conventional VWF:Act cutoff of 50 IU/dLwith ususual bleeding, similar to inherited VWD, could be considered for clinical practice.

Acquired platelet function defects (PFDs) with MPN have frequently been described but testing in clinical practice is less common because of limited availability and difficulty in interpreting the results [32,42]. PFDs arise from the heterogeneous and dynamic alteration in platelet population derived either from the malignant hematopoietic clone or secondary to enhanced platelet activation in vivo [42,43,44]. The numerous abnormalities range from hypofunction [32,42,45,46] to hyperfunction [47,48,49,50,51]. Individual assessment of responsiveness to the dose and frequency of aspirin to prevent thrombosis has been proposed in some research studies [32,52]. Clinical testing of platelet function should be considered in patients presenting acutely with excessive bleeding or immediately prior to surgery to guide management. If there is enough time prior to elective surgery, cytoreduction therapy is usually often considered but does not always correct the platelet function defect or aVWS in MPNs [37,53,54].

### 2.4. Management of Bleeding

As there is a lack of randomized clinical trials, management on MPN-associated bleeding is a shared decision between the physician and patient. Control of the MPN with cytoreductive therapy can sometimes assist in correction of the haemostatic defect of aVWS or platelet function [37,53,54]. It is recommended that screening for aVWS is performed if the platelet count >1000 × 10^9^/L and it seems reasonable to screen patients with a platelet count >450 × 10^9^/L prior to surgery [5,53]. For each surgical intervention, we must consider a risk-adapted approach, especially if there are preexisting bleeding symptoms, the surgery is high risk for bleeding or the preoperative VWF:Act ratio is below <50% [10,41]. Frequently, desmopressin (DDAVP) is used for aVWS and PFD, VWF concentrates are used for aVWS, tranexamic acid is used for aVWS and PFD, and single-donor platelet transfusion is used if the bleeding is suspected to be caused by qualitative platelet dysfunction. For elective procedures, cessation of anti-thrombotic medication is recommended based on its half-life. For VTE prophylaxis post-surgery, mechanical measures can initially be used until hemostasis is secure and the patient can transition to extended duration aspirin or LMWH at a preventative dose [10,53]. Multidisciplinary collaboration between the hematologist, surgeon and anesthetists is essential for minimizing excess bleeding and thrombosis in MPN patients.

**Case Presentation (Continued):** In this case, with a recently acquired bleeding diathesis, differentiating between a reactive versus clonal cause of thrombocytosis is challenging especially in the setting of iron deficiency anemia. Following intravenous iron, her thrombocytosis persisted raising the possibility of an MPN. Further investigation confirmed the JAK2V617F mutation and mild splenomegaly (15 cm in span) consistent with ET. She underwent further haemostatic testing, and she was found to have aVWS with a VWF: Ag of 45 IU/dL (normal range > 50 IU/dL) and VWF: Act of 22 IU/dL (normal range > 50 IU/dL) with a VWF: Act to VWF: Ag ratio of 0.49 and normal platelet aggregation studies consistent with aVWS. She started on weekly pegylated interferon alfa-2a (INF) with normalization of her platelet count and correction of her aVWS. Although the menorrhagia improved following INF, an endometrial oblation was also subsequently performed that eliminated her HMB.

## 3. Thrombosis in Women with MPNs

**Case Presentation 2:** A 31-year-old woman with no prior medical history presented with jaundice and abdominal pain with distension for 1 month. Computed tomography of the abdomen revealed massive ascites and non-visualized hepatic vein, consistent with Budd–Chiari syndrome. CBC showed hemoglobin of 16.6 g/dL, white blood cell count of 21.7 × 10^6^/L, and platelet count of 245 × 10^9^/L. Bone marrow study was compatible with polycythemia vera. JAK2 V617F was positive. Hematology was consulted for the long-term management of this patient.

Thrombosis is a common complication in MPNs, affecting approximately 20% of patients at the time of diagnosis [4]. Compared to matched controls, individual with MPNs have significantly higher rates of thrombosis (3-month HR 3.0: 95% [CI 2.7–3.4] for arterial thrombosis, HR 9.7: 95% [CI 7.8–12] for venous thrombosis, and a striking HR 81.1: 95% CI [22.0–300.0] for splanchnic vein thrombosis [55]. Not only is it common, but thrombosis also contributes significantly to the mortality of MPN patients. Individuals with MPN face a twofold increased risk of death compared to the general population, with cardiovascular and cerebrovascular diseases accounting for approximately 20% of mortality [56]. Notably, younger patients (<59 years) with MPNs have an elevated probability of dying from cardiovascular disease (cerebrovascular HR 5.1: 95% CI [2.8–9.4], cardiovascular HR 2.2: 95% CI [1.6–3.1]) compared to age-matched controls [56]. The mechanisms of thrombosis in MPN are complex and multifactorial [57]. Erythrocytes exhibit increased adhesion to the endothelium, independent of erythropoietin [58]. Elevated inflammatory cytokines lead to activation of neutrophils, platelets, and endothelial cells, promoting the formation of neutrophil–platelet aggregates and neutrophil extracellular traps [57]. Additionally, JAK2-mutated vascular endothelial cells demonstrated proinflammatory and pro-adhesive phenotypes [57].

Although the overall rates of thrombosis in MPNs are similar across sexes [59], women with MPNs exhibit a distinct thrombosis profile compared to men [60]. The analysis of 1638 patients with PV revealed a higher frequency of microcirculatory disturbances and venous thrombosis in women [60], whereas myocardial infarction was more frequent in men [60]. Among venous thrombosis cases, 16.7% were splanchnic vein thrombosis, which commonly presented concurrently with or preceded the diagnosis of MPNs in women. Notably, women constitute the majority of retrospective cohorts investigating MPN-associated splanchnic vein thrombosis [61,62,63]. The underlying reasons for these sex-specific differences remain unclear and warrant further investigation. Interestingly, most cases of MPN-associated splanchnic vein thrombosis were not associated with oral contraceptive or hormonal use, suggesting that estrogen may not be the primary mechanism underlying this phenomenon [63].

This case, presenting with one form of splanchnic vein thrombosis with Budd–Chiari syndrome, highlights the tendency of this condition to predominantly affect young women with MPNs [15]. MPNs are identified in approximately 40% of patients with Budd–Chiari syndrome, with 17% of these cases occurring in the absence of typical hematologic features of MPN [64]. Given the therapeutic implications, routine testing for MPN-driver mutations, *JAK2* V617F, CALR, and MPL sequentially, should be performed in patients with splanchnic vein thrombosis, particularly in those without obvious provoking factors, regardless of blood count abnormalities [65].

Management of MPN-associated splanchnic vein thrombosis is similar across sexes, with anticoagulation being the cornerstone of treatment. Life-long vitamin K antagonist (VKA) is recommended, with the aim to prevent recurrent thrombosis and preserve liver function [66]. A meta-analysis reported a recurrence rate of thrombosis of 1.4 per 100 patient-years and a major bleeding rate of 2.8 per 100 patient-years associated with anticoagulation therapy [67]. Accumulating evidence suggests that DOACs are safe alternatives and may be considered in patients with a low bleeding risk and normal liver function [68]. However, comparative data on the efficacy and safety of different anticoagulants in this population are lacking. In addition to anticoagulation, cytoreductive therapy is indicated to control disease activity and reduce thrombotic risk, particularly in patients with high-risk MPN features or inadequate hematologic control. Lifestyle modifications, including adopting a healthy dietary pattern, maintaining healthy body weight, smoking cessation, and increasing physical activity, may help modulate systemic inflammation and potentially modify thrombotic risk [69]. Further studies are needed to evaluate the impact of these lifestyle changes on MPN outcomes.

During the treatment of MPN, patients should be advised to use effective contraception methods to prevent unplanned pregnancies. Estrogen-containing oral contraceptives should be avoided, as their use has been associated with a threefold increase in venous thrombosis risk and a fivefold increase in splanchnic vein thrombosis among MPN patients [70]. Alternative contraceptive methods, such as progestin-only contraceptives, intrauterine devices (IUDs), or implants, should be considered and discussed with the patient.

Case presentation (continued): The patient was initially treated with warfarin and received regular phlebotomy with a hematocrit threshold of 45%. However, despite undergoing a transjugular intrahepatic portosystemic shunt (TIPS) procedure, she later progressed to Child–Pugh class C cirrhosis, which complicated her treatment due to an increased risk of bleeding and difficulties in monitoring anticoagulation therapy. Consequently, her anticoagulation regimen was switched to enoxaparin 1 mg/kg twice daily. The patient underwent a liver transplant as definitive treatment for her advanced liver disease.

## 4. MPNs and Pregnancy

**Case presentation (continued):** At age 45 years old, the above-mentioned patient presented with new-onset nausea and vomiting and amenorrhea. Upon evaluation, she was found to be pregnant, with a gestational age of 19 weeks. She subsequently visited the hematology clinic for counseling regarding the management of her pregnancy in the context of her underlying MPN and prior medical history.

The incidence of MPN-related pregnancies is estimated to be 3–12 per 100,000 pregnancies [23,71]. MPNs can significantly affect fertility and pregnancy outcomes. Women with MPNs are at increased risk for complications such as thrombosis, recurrent pregnancy loss, preterm delivery, placental insufficiency, preeclampsia and post-partum hemorrhage [7]. Concerns regarding teratogenicity and fetal toxicity from MPN-directed therapies add complexity to pregnancy planning and management. Notably, recurrent miscarriage can sometimes be the first indication of an underlying MPN, leading to its diagnosis. Similarly, pregnancy itself can influence the course of MPNs. The perinatal period often involves invasive procedures, which heightens the potential for bleeding complications. Additionally, the physiological changes in blood counts during pregnancy can affect hematologic management, such as adjusting the hematocrit threshold for optimal control in PV. These challenges underscore the need for multidisciplinary care to optimize outcomes for both mother and fetus.

### 4.1. MPN and Fertility

A common concern among women of childbearing age with MPN is the potential impact on fertility and pregnancy outcomes. While current evidence is limited, emerging studies offer some valuable insights. In a population-based study that compared the childbirth rate of 1141 women with MPN to 4565 age-matched controls, women with MPN had a 22% lower likelihood of successfully undergoing childbirth. Interestingly, while childbirth rates were significantly reduced in women with PV and PMF, those with ET were comparable to controls. Women with MPN also had fewer children on average (1.82 vs. 2.01) and a higher prevalence of previous stillbirths at diagnosis [72]. Additionally, a systematic review and meta-analysis of 22 observational studies reported an overall live birth rate of 71.3%, with rates of 71.1% for ET and 66.7% in PV [73]. These figures are slightly lower than the approximate 80% live birth rate observed in the general population [74]. It remains unclear whether MPN, particularly PV and PMF, have a direct biological impact on fertility, or if women with MPN chose not to conceive due to the challenges associated with the diagnosis and its treatment. However, the data suggest that with optimized care, successful pregnancy is achievable for most women with MPN.

### 4.2. Pregnancy Outcomes and Their Prediction in MPN

In a population-based study comparing 342 MPN pregnancies with control pregnancies (matched for age, calendar year, and parity), MPN pregnancies were associated with significantly increased preterm birth (14% vs. 4%), low birth weight (<2500 g, 10% vs. 4%), and cesarean section (32% vs. 17%) [23]. Preclampsia was identified as one of the most common obstetric complications, with a pooled incidence of 3.1% reported in a systematic review and meta-analysis [73].

Thrombotic complications were consistently noted to be more frequent in MPN pregnancies compared to the general population. A meta-analysis of 21 retrospective studies, encompassing 756 pregnancies in women with ET, reported VTE rates of 1.3% during the antepartum period and 1.8% during the postpartum period [75]. Notably, the risk was higher in pregnancies where low-molecular-weight heparin (LMWH) was not administered, with VTE rates increasing to 2.5% antepartum and 4.4% postpartum [75], supporting the use of LMWH prophylaxis in ET during the postpartum period. A more recent meta-analysis that included all MPN subtypes reported a pooled VTE incidence of 1.5% [73]. Additionally, one study found a thrombosis rate of 1% in MPN pregnancies compared to 0% in controls [23]. A series of 129 PV pregnancy reported a higher thrombosis rate of 3.1% [76].

Bleeding complications are another significant concern in MPN pregnancies. A trend toward increased bleeding risk was observed in one study, with rates of 14% in MPN pregnancies compared to 9% in controls [23]. In PV pregnancies, bleeding was the most frequently reported complication, occurring in 15% of cases [71]. A meta-analysis reported pooled incidences of postpartum hemorrhage at 1.5% and other bleeding events at 1.1% [73]. The variability in thrombosis and bleeding rates across studies may reflect differing criteria for defining thrombosis and bleeding endpoints, emphasizing the need for standardized outcome measures in this patient population.

Ideally, patients with predictive factors for pregnancy-related complications should be stratified and managed accordingly. However, the identification of reliable predictive factors remains an area requiring further investigation. Current thrombotic risk stratification models for MPN, such as components of the IPSET score, are not applicable for predicting complications in pregnancy [77]. In a study evaluating 121 pregnancies in women with ET, a history of prior pregnancy loss was significantly associated with subsequent pregnancy complications, whereas factors such as JAK2 V617F and CALR mutation status, maternal age, and pre-pregnancy blood counts showed no significant association [78]. Additionally, diabetes mellitus was associated with fetal loss and preclampsia in ET pregnancies [77]. The impact of the JAK2 V617F mutation on pregnancy outcomes has been inconclusive, with earlier studies suggesting a correlation between the mutation and pregnancy complications [79,80] while more recent studies found no significant association [73,77,78].

### 4.3. Management of MPN in Pregnancy

**Figure 3:**
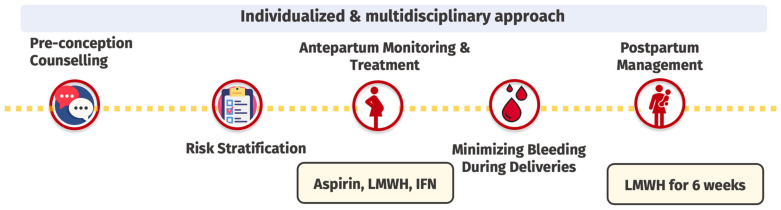
Framework for the management of myeloproliferative neoplasm in pregnancy. LMWH: low-molecular-weight heparin, INF: interferon, wk: weeks.

With the absence of randomized controlled trials specific to this population, the management of MPN during pregnancy is predominantly guided by expert opinions, observational data, and extrapolations from management strategies used in other high-risk pregnancies [7]. We propose a step-wise management approach for MPN-related pregnancy as follows (Figure 3):

#### 4.3.1. Preconception Counseling

Discussions about pregnancy should be an integral part of the routine follow-up for women with MPN who are of reproductive age. For a small subset of patients, such as those with high-risk overt myelofibrosis characterized by severe cytopenia and significant symptoms requiring intensive treatment, pregnancy may need to be discouraged or deferred. In these cases, appropriate contraception methods should be recommended to ensure effective family planning and discussion about further therapy such as allogeneic stem cell transplantation considered.

For patients desiring pregnancy, comprehensive counseling is essential to address the potential risks of maternal and fetal complications. This includes discussing the possibility of thrombotic and bleeding events, as well as other pregnancy-related complications associated with MPN. Achieving optimal control of MPN and other comorbidities for maternal and fetal complications such as diabetes or essential hypertension before conception is crucial. Equally important is transitioning from teratogenic medications to those considered safe during pregnancy. For instance, hydroxyurea should be discontinued and replaced with INF for at least 3 months before conception, while warfarin or DOACs should be switched to LMWH to minimize risks to the fetus. Anagrelide and *JAK2* inhibitors such as ruxolitinib are not approved for use in pregnancy. An early interdisciplinary approach is critical, involving close collaboration between experienced obstetricians, hematologists, and, when necessary, other specialists.

#### 4.3.2. Risk Stratification

Risk stratification models specifically tailored for MPN pregnancies are not available. However, certain clinical factors can help identify high-risk patients (Table 2). Women with a previous history of thrombosis or bleeding should be classified as high-risk. Additionally, patients with a prior history of pregnancy complications—such as those outlined in antiphospholipid syndrome criteria—are also considered high-risk. These patients benefit from closer monitoring and targeted interventions, including the use of interferon therapy, to manage their condition and improve pregnancy outcomes

#### 4.3.3. Antepartum Monitoring and Treatment

In all MPN-pregnancies, low-dose aspirin should be recommended throughout pregnancy, as it has been shown to improve outcomes with minimal bleeding risk. Studies have demonstrated that aspirin use in MPN pregnancies was associated with a significantly higher live birth rate (unadjusted odds ratio, OR 8.6; 95% CI [4.0–18.1]) [73], a lower rate of unintentional fetal loss (45% vs. 14%) [77], a reduced risks of pregnancy complications (OR 0.29; 90% CI [0.12–0.66] [78], and a lower risk of thrombosis (0% vs. 3%) [77]. A recent retrospective study reported the safe use of aspirin in 67% of patients with aVWS without maternal hemorrhage [77] but advised that it must be stopped soon before delivery. However, aspirin should generally be avoided in patients with extreme thrombocytosis and acquired von Willebrand syndrome due to an increased risk of bleeding.

In patients with PV, therapeutic phlebotomy can be continued or initiated as indicated during pregnancy. Given the physiological decline in hemoglobin levels due to plasma volume expansion, the target hematocrit for phlebotomy should be adjusted based on gestational age, typically ranging from 37% to 41%. Caution must to be taken against iron supplementation in this population, as it can lead to an increase in hematocrit levels. Fetal monitoring, including placental flow scans, is recommended at gestational weeks 20, 32, and 36, with more frequent intervals if complications are suspected. Additional thrombotic risk factors, such as immobility and hyperemesis, should be monitored and managed appropriately.

Patients with high-risk features may benefit from interferon (IFN) and/or therapeutic LMWH. Treatment with IFN has been associated with increased odds of live births in MPN patients (OR 9.7; 95% CI [2.3–41.0]) [73]. Although further prospective clinical trials are needed to confirm these findings, IFN is often considered in MPN patients requiring cytoreductive therapy, those with a history of thrombosis, or those with prior pregnancy complications. Pegylated interferon alpha-2a has been shown to be a safe and effective alternative in pregnant women with ET [81]. In patients with prior history of thrombosis or a pre-existing indication for anticoagulation, a preventive dose or therapeutic weight-based dose of LMWH is indicated. However, the benefit of LMWH in preventing non-thrombotic pregnancy complications remains uncertain. A prior meta-analysis found no improvement in live birth rates among MPN patients treated with heparin compared to those managed with aspirin alone or observation [73]. Similarly, a recent randomized control trial found no benefit of LMWH in improving live birth rates in patients with inherited thrombophilia and recurrent pregnancy loss [82]. Antepartum bleeding risk associated with LMWH use in MPN is approximately 4% [75]. The decision to use LMWH in patients without a history of thrombosis should be individualized, with a careful risk–benefit discussion. Identifying patient characteristics that may predict a benefit from LMWH remains an important area for future research.

#### 4.3.4. Prevention of Peripartum Bleeding

The peripartum period carries the highest risk of bleeding, particularly due to procedure-related factors. Women with MPN are at an increased risk of delivering via cesarean section [23]; however, the mode of delivery should primarily be determined based on obstetric indications and complications rather than the presence of MPN alone.

For women receiving therapeutic LMWH, it is recommended to discontinue LMWH at least 24 h prior to delivery to reduce the risk of bleeding. Similarly, low-dose aspirin should be stopped by the 37th week of gestation or 7 days before the expected onset of labor to minimize bleeding risk during delivery.

Platelet counts should be closely monitored in the peripartum period, particularly in patients with extreme thrombocytosis (>1000–1500 × 10^9^/L), as these patients are at increased risk of aVWS, which can further elevate the bleeding risk.

#### 4.3.5. Postpartum Management

Prophylactic LMWH is recommended during the 6-week postpartum period for women with MPN due to the elevated risk of higher VTE, estimated at 4.4%, which can be effectively mitigated with the use of LMWH [75]. However, the additional benefit of LMWH over aspirin alone during this period is not well established, and whether patients should continue aspirin, LMWH, or a combination of both in the postpartum period remains unclear, highlighting the need for further research. Aspirin and IFN can be safely continued during breastfeeding for patients with high-risk MPN. Additionally, effective contraception should be recommended to prevent unintended pregnancies.

**Case presentation (continued):** Given her history of Budd–Chiari syndrome, the patient was considered to have a high-risk MPN pregnancy. Low-dose aspirin was initiated, while enoxaparin at a therapeutic dose and phlebotomy were continued throughout pregnancy. IFN was considered but not given due to limited access and control of her hematocrit with her venesections. Unfortunately, her pregnancy was complicated with severe preeclampsia and low birth weight requiring urgent caesarian section and cessation of LMWH and aspirin. She also developed aVWS during the peripartum period with postpartum hematoma requiring evacuation. During the postpartum period, once hemostasis was secure, she was restarted with a prophylactic dose of LMWH safely.

## 5. Conclusions

Women with MPN face unique challenges throughout the course of their disease and require a tailored, multidisciplinary approach to effectively address these issues (Table 3).

As the evidence to guide practice in MPN is limited, in the illustrated case vignettes, we reviewed and highlighted the practical management of thrombotic, bleeding, and pregnancy complications in women with MPN. With appropriate care and management, it is reassuring that most women with MPN can achieve successful pregnancies. Future research should focus on identifying MPN-specific predictive factors for women’s health and pregnancy complications and evaluating the benefits of individualized treatments to further improve outcomes.

## Figures and Tables

**Table 1:** Comparison of sex-specific differences in myeloproliferative neoplasms [11,12,13,14,15].

Female patients	ET is more prevalent compared to PV and MF. Higher symptom burden (abdominal pain, microvascular symptoms (headache, fatigue, insomnia, concentration difficulties, dizziness). Lower *JAK2* V617F allele load independent of age or disease duration. Fewer non driver somatic mutations such as *KRAS*, *NRAS*, *EZH2*, and *CBL*. Higher rates of venous thromboembolism (DVT/PE) and unusual thrombosis (cerebral or splanchnic vein) but not arterial thrombosis.
Male patients	Shorter overall survival. Higher likelihood of progression to secondary MF or AML.

ET, essential thrombocythemia; PV, polycythemia vera; MF, myelofibrosis; DVT, deep vein thrombosis; PE, pulmonary embolism; AML, acute myeloid leukaemia.

**Table 2:** Features of high-risk MPN pregnancy.

High-risk MPN	History of thrombosis History of bleeding
History of pregnancy morbidity	Recurrent unexplained early pregnancy loss (gestation age < 10 weeks) Fetal loss (early: 10–16 weeks; late: 16–34 weeks) Preeclampsia Placental insufficiency (intra-uterine growth restriction, still-birth, placental abruption, or oligohydramnios) Prematurity (gestation age < 34 weeks)

MPN, myeloproliferative neoplasm.

**Table 3:** Summary of sex-specific challenges in MPN and management strategies.

Challenges		Management Approaches
Bleeding	Heavy menstrual bleeding due to aVWS and platelet dysfunction Increased risk of surgical and post-partum hemorrhage	Screen for aVWS in women with high platelet counts Consider cytoreductive therapy Use tranexamic acid, desmopressin, or platelet transfusion as needed
Thrombosis	Higher frequency of venous thrombosis, especially splanchnic vein thrombosis in women	Avoidance of estrogen-based contraceptives High index of suspicion for MPN in splanchnic vein thrombosis Anticoagulation approach similar to men, with additional concerns for menorrhagia
Fertility	Lower likelihood of achieving childbirth than the general population (in PV and PMF) Lower number of children and high prevalence of miscarriages and stillbirths	Preconception counseling and multidisciplinary care Optimize disease control before conception Avoid teratogenic treatments during conception (e.g., hydroxyurea or anagrelide)
Pregnancy and postpartum	Increased risk of obstetric complications including pre-term birth and low birth weight Increased risk of thrombosis during pregnancy and postpartum period Increased bleeding risk, especially around delivery	Preconception counseling and multidisciplinary care Low-dose aspirin and LMWH Cytoreduction with phlebotomy or interferon

aVWS: acquired von Willebrand syndrome. PV; polycythemia vera. PMF; primary myelofibrosis, MPN; myeloproliferative neoplasm. LMWH; low-molecular-weight heparin.
